# Idiopathic Non Cirrhotic Portal Hypertension and Spleno-Portal Axis Abnormalities in Patients with Severe Primary Antibody Deficiencies

**DOI:** 10.1155/2014/672458

**Published:** 2014-03-31

**Authors:** Federica Pulvirenti, Ilaria Pentassuglio, Cinzia Milito, Michele Valente, Adriano De Santis, Valentina Conti, Giulia d'Amati, Oliviero Riggio, Isabella Quinti

**Affiliations:** ^1^Department of Molecular Medicine, Sapienza University of Rome, Viale dell'Università 37, 00186 Rome, Italy; ^2^Gastroenterology, Department of Clinical Medicine, Sapienza University of Rome, Rome, Italy; ^3^Department of Experimental Medicine and Pathology, Sapienza University of Rome, Rome, Italy

## Abstract

*Background and Aim*. Portal hypertension has been reported in association with acquired and primary immune deficiencies without a comprehensive description of associated spleno-portal axis abnormalities. Pathological mechanisms are poorly defined. *Methods*. Observational, single centre study with the aim of assessing the prevalence of spleno-portal axis abnormalities in an unselected cohort of 123 patients with primary antibody deficiencies and without known causes of liver diseases regularly followed up for a mean time of 18 ± 14 years. A cumulative period of 1867 patients-year was analysed. Clinical and immunological data, abdominal ultrasounds, CT scans, and endoscopy features were included in the analysis. *Results*. Twenty-five percent of patients with primary antibody deficiencies had signs of portal vein enlargement but only 4% of them had portal hypertension, with portal systemic collaterals. Liver biopsies showed liver sinusoids congestive dilatation, endothelization, and micronodularity fulfilling the criteria for noncirrhotic portal hypertension. Patients with portal vein enlargement had severe clinical and immunological phenotypes. *Conclusions*. In primary antibody deficient patients, infections, inflammations, splenomegaly, increased blood venous flow, and lymphocyte abnormalities contribute to establishment of liver damage possibly leading to noncirrhotic portal hypertension. Patients with primary antibody deficiency should be considered a good model to give insight into the pathological mechanisms underlying noncirrhotic portal hypertension.

## 1. Introduction 

Common variable immunodeficiency (CVID) and congenital X-linked agammaglobulinemia (XLA) are the most common symptomatic primary antibody-deficiency syndrome (PAD). CVIDs are a heterogeneous group of hypogammaglobulinemia deficiencies of unknown aetiology, frequently diagnosed in adults [1]. XLA is inherited as an X-linked recessive trait and caused by B cell specific tyrosine-kinase (BTK) gene mutations resulting in a bone marrow defective B cell differentiation [2]. CVID patients are affected by recurrent infections in the airways and gastrointestinal tract, causing bronchitis, pneumonia, otitis, chronic sinusitis, and gastroenteritis. Autoimmune disorders and granulomatous diseases might also be a part of PAD-associated inflammatory diseases [3–5]. The liver diseases in these conditions are poorly defined. Before HCV screening test and viral inactivating steps in immunoglobulin (Ig) production for replacement treatment, RNA viruses were recognized as causes of acute and chronic liver diseases [6]. Later, a condition of nodular regenerative hyperplasia (NRH) has been described [7–11]. More recently, a condition known as idiopathic noncirrhotic portal hypertension (INCPH) whose morphological substrate is the presence of NRH was found to be associated with many immunological disorders [12, 13]. In PAD patients selected because of liver abnormalities, a high prevalence of cholestasis (65%) and portal hypertension (50%) was found. Liver biopsies revealed nonfibrosing architectural abnormalities consistent with NRH and anomalies of portal vessels [8]. Here, we describe the prevalence of spleno-portal axis abnormalities in an unselected cohort of patients of PAD without known causes of liver diseases.

## 2. Methods

### 2.1. Study Design and Participants

It is an observational, single centre study. An unselected cohort of 123 patients with a PAD diagnosis (117 affected by CVID and 6 by XLA) based on the current diagnostic criteria of the European Society for Immune Deficiencies/Pan-American Group for Immune Deficiencies (ESID/PAGID) [14] were included in the study. All the 123 patients with PAD have been regularly followed up in our Referral Centre for Primary Immune Deficiencies. According to international guidelines for recipients of plasma derivatives (polyvalent immunoglobulins) every year all patients under replacement treatment underwent HIV RNA, HCV RNA, and HBsAg detection. All patients were HIV negative. Five CVID patients with concomitant HCV or HBV infection and one with histologically proven primary biliary cirrhosis were excluded. One patient with CVID was excluded for being affected by primary biliary cirrhosis, histologically proven. The mean follow-up time of the 117 patients (111 CVID and 6 XLA) analysed for the study was 18 ± 14 years. A cumulative period of 1867 patients-year was included in the analysis. Daily alcohol consumption was less than 30 g for men and 20 g for women. None of the patients had a history of intravenous drug abuse. All participants provided written informed consent.

### 2.2. Data Sources/Measurement

Detailed information including personal data, pedigree, and age at diagnosis, immunological data, and clinical manifestations such as pneumonia, gastroenteritis, and autoimmune diseases were collected from medical files. All data were processed in a database and sent to CINECA, an interuniversity computing centre responsible for processing and analysing the data. The following data were collected for each patient every 4 months following the Italian PAD guidelines (http://www.aieop.org/) [15]: platelet (PLT), red blood cell (RBC), and white blood cell (WBC) counts; chemistries: aspartate aminotransferase (AST), alanine aminotransferase (ALT), alkaline phosphatases (AP), gamma-glutamyl transferase (GGT), total bilirubin, serum albumin, and prothrombin time. Blood samples were collected once a year and tested by an enzyme immunoassay technique (EIA) for detection of hepatitis B surface antigen (HBsAg); HCV-RNA was tested by using nested-PCR assays. Chest computerized tomography (CT) scans were performed every four years according to the Italian guidelines. Upper gastrointestinal endoscopy with gastric and duodenal biopsies was performed every two years due to the increased risk of CVID-associated gastric cancer and lymphoproliferative diseases [16]. Abdominal ultrasonography (US) [17] was performed every 2–4 years in all patients older than 18 years of age by the same investigator working in our centre. Peripheral blood lymphocyte immunophenotyping by flow cytometry was available for 64 patients. Liver biopsy was performed in the five patients (patients numbers 2, 43, 55, 59, and 72 in Supplementary Table 1; see Supplementary Material available online at http://dx.doi.org/10.1155/2014/672458) with esophageal varices or portosystemic collateral vessels to exclude liver cirrhosis. Histological features were viewed by two expert pathologists.

### 2.3. Definitions Adopted

*Liver abnormalities*: liver enzymes are aspartate aminotransferase (AST) > 40 UI/L, alanine aminotransferase (ALT) > 40 UI/L, alkaline phosphatase (AP) > 129 UI/L, gamma glutamyl transferase (GGT) > 61 UI/L, and total bilirubin > 12 mol/L for at least for six consecutive months. Cholestasis: elevated AP and/or elevated GGT.* Splenomegaly* occurs when spleen longitudinal axis is ≥ 12 cm in at least two consecutive exams.* Portal vein enlargement *occurs when portal vein diameter is  > 13 mm in at least two consecutive ultrasound examinations (measurements of the portal vein were obtained at its broadest point just distal to the union of the splenic and superior mesenteric veins).* Spleno-portal axis abnormalities* are association between splenomegaly and portal vein enlargement. Noncirrhotic portal hypertension is diagnosed according to the criteria defined by Schouten et al. [12].* Bronchiectasis* is shown as localized, irreversible dilation of part of the bronchial tree by CT scan.* Pneumonia*'s symptoms and signs are consistent with an acute lower respiratory tract infection associated with new radiographic shadowing for which there was no other explanation (e.g., not pulmonary oedema or infarction) [18].* Recurrent gastroenteritis* has three or more episodes in a year of nausea, vomiting, diarrhoea, and abdominal cramps that could be accompanied by fever.* Lymphoid nodular hyperplasia (LNH)* is the presence of lymphoid aggregates found in the small intestine identified by intestinal biopsy*. Autoimmune manifestations* are diagnosed according to established criteria for rheumatoid arthritis, systemic lupus erythematosus, thyroiditis, Sjögren syndrome, and autoimmune cytopenia.

*Histological Evaluation*. Liver biopsy specimens were fixed in formalin, paraffin-embedded, and stained with hematoxylin eosin, Masson's trichrome, and picrosirius red and silver stain for reticulum. The slides were viewed under light microscopy. The size of the liver biopsy and the number of portal tracts were recorded. Intrahepatic lymphocyte immunostaining was performed on paraffin sections using monoclonal antibodies directed against CD3 (Dako, Glostrup, Denmark). Sinusoid endothelization was assessed with CD34 immunostaining with specific antibodies (Dako, Glostrup, Denmark).

*Statistical Methods*. The Kruskal-Wallis one-way analysis of variance was used for comparison of data. Comparison between single parameters was assessed by simple linear regression analysis. Comparison between patients' groups was performed by contingency tables. Statistical analysis was performed with StatView software or GraphPad Prism software. A *P* value equal or less than 0.05 was considered to be statistically significant.

## 3. Results

One hundred and seventeen patients, 111 affected by CVID and 6 by XLA, were included in the analysis. The mean age was 49 ± 15 years. The mean follow-up time was 18 ± 14 years. The characteristics of the patients are summarized in Table 1. Detailed data are provided in Supplementary Table 1. A cumulative period of 1867 patients-year was included in the analysis. A total of 442 abdominal ultrasounds have been reevaluated for this study analysis.

### 3.1. PAD Patients with Spleno-Portal Axis Abnormalities Have a Severe Clinical Phenotype

**Figure 1:**
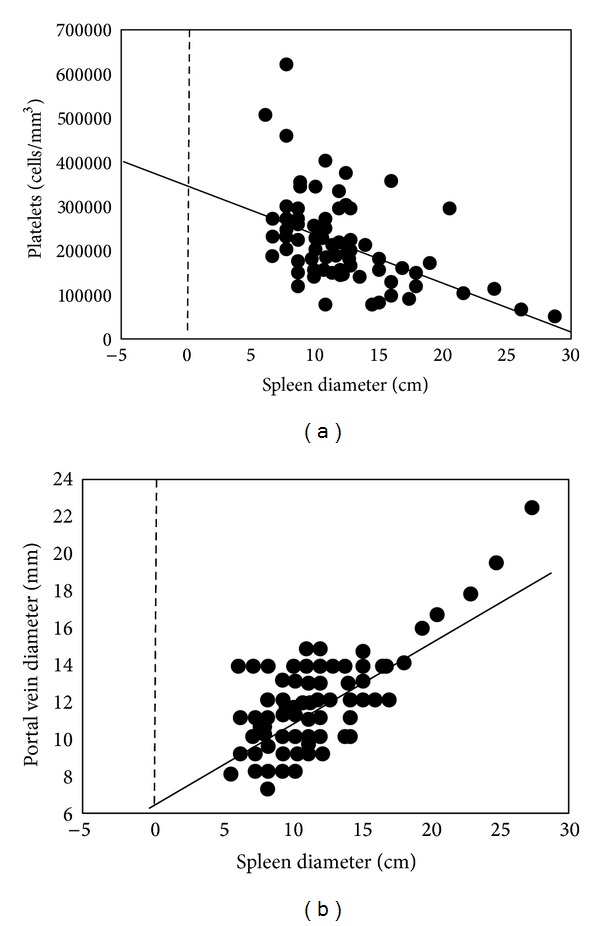
Spleen diameter, portal vein diameter, and platelets count in the 117 PAD patients. Regression analysis showed the correlation between spleen diameter and platelets counts and between spleen and portal vein diameters.

**Figure 2:**
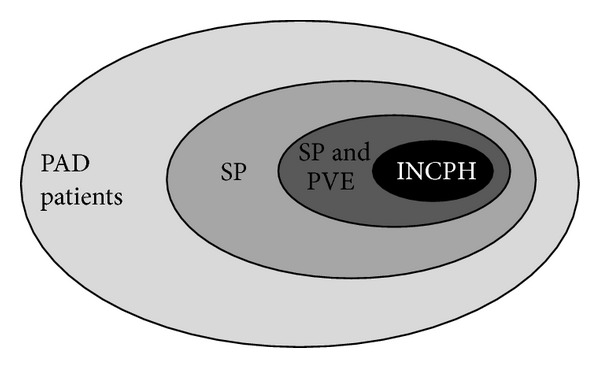
Distribution of PAD patients according to the abdominal ultrasound findings. PAD: primary antibody deficiencies; SP: PAD patients with isolated splenomegaly; SP and PVE: PAD patients with splenomegaly and portal vein enlargement; INCPH: PAD patients with idiopathic noncirrhotic portal hypertension.

There is a general agreement that splenomegaly is a common feature in PAD but its consequences are not well understood [19]. Here, we confirmed our previous data [20] showing a spleen enlargement in 71/117 patients (61%) of our cohort. Spleen diameter was highly correlated with portal vein diameter (*R*2 = 0.5; *P* < 0.0001) (Figure 1) suggesting that an increased splenic venous flow secondary to splenomegaly could contribute to determining a condition of portal superflux. At ultrasound, 30 out of 117 patients (25.6%) had signs of portal vein enlargement but only 1/6 of these had portal hypertension/INCPH, with portal systemic collaterals (Figure 2). Longitudinal assessment of abdominal ultrasounds demonstrated that portal vein enlargement and splenomegaly slightly increased in the observation period (Supplementary Figure 1). In the subgroup of patients without spleno-axis abnormalities, spleen and portal vein diameters remained within the normal ranges during the observational period. Diameters of portal vein and liver were also significantly associated (*R*2 = 0.34, *P* = 0.0009). Spleno-portal axis abnormalities were invariably associated with a more severe PAD phenotype (Table 2) with a higher prevalence of bronchiectasis (*P* = 0.05), gastroenteritis (*P* = 0.0002), lymphoid nodular hyperplasia (*P* = 0.009), and autoimmune manifestations (*P* = 0.03). The inverse correlation between platelet count and spleen diameter (*R*2 = 0.22; *P* < 0.0001) suggests a mechanism of splenic sequestration as the main cause of thrombocytopenia in PAD patients (Figure 1).

### 3.2. INCPH in PAD Patients

**Figure 3:**
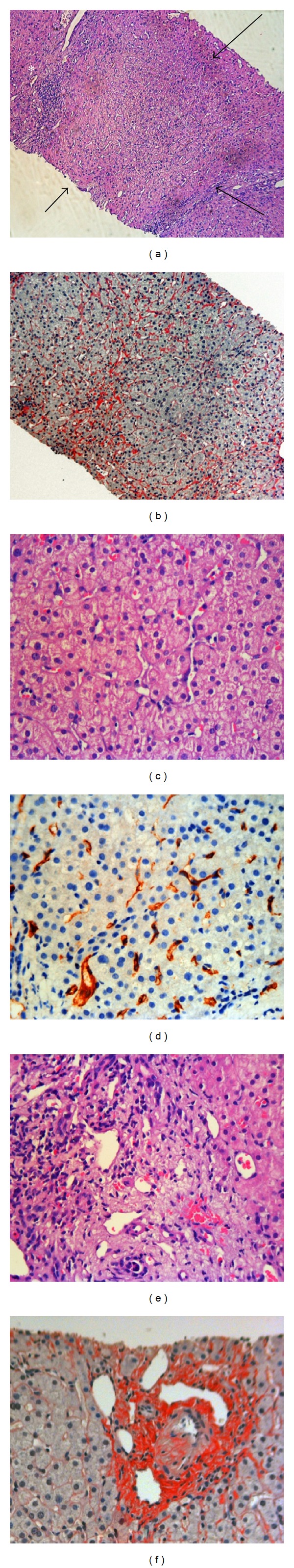
Liver biopsy from patient 2 showing features of nodular regenerative hyperplasia. (a-b) Liver parenchyma shows nodules with expanded liver plates surrounded by a rim of atrophic hepatocytes (arrows), in absence of fibrous septa. Portal lymphohistiocytic inflammatory infiltrate with interface activity (white asterisk) is also present ((a) hematoxylin and eosin stain, original magnification 10x; (b) Sirius Red stain, 10x). Congested and dilated sinusoids (megasinusoids) are shown in (c) and (d) ((c) hematoxylin and eosin stain, original magnification 20x; (d) CD34 immunostaining, original magnification 20x); (e) aberrant vascular structures within the portal tract (hematoxylin and eosin, original magnification 20x); (f) fibrotic portal tracts (Sirius Red stain, original magnification 40x).

Thirty patients (28 CVID, 2 XLA) had portal vein enlargement detected by ultrasounds, an indirect index of portal hypertension. Four of these patients (3 CVID and 1 XLA) had esophageal varices (3 small and one large) at upper gastrointestinal endoscopy and one patient had portal vein collaterals detected by CT scan. None of the patients had portal hypertensive gastropathy. These five patients underwent liver biopsy, which excluded cirrhosis and thus they fulfilled the diagnostic criteria for INCPH [12]. In the remaining 25 patients with portal vein enlargement without other clinical or radiological signs of portal hypertension the liver biopsy was not performed for ethical reasons. In patient with INCPH, histological findings consisted of liver sinusoidal congestive dilatation, presence of paraportal shunt vessels in the portal tract, and sinusoidal endothelization. Parenchymal changes consisted of a micronodular transformation with nodules surrounded by an atrophic rim of hepatocytes in absence of fibrosis. Mild portal lymphohistiocytic inflammatory infiltrate was also observed. Representative aspects of architectural changes and sinusoidal inflammation observed in a PAD patient (patient number 2) are shown in Figure 3. In blood chemistry, mainly signs of cholestasis were found, common in liver diseases [21]. All 5 patients (100%) who fulfilled the criteria for INCPH had abnormalities in liver enzymes: 4 had signs of cholestasis and 1 showed an increase of transaminase levels (Supplementary Table 1). Signs of cholestasis in the absence of INCPH were also detected in 1 patient with portal vein enlargement and splenomegaly, in 3 patients with isolated splenomegaly, and in 1 patient without spleno-portal abnormalities. Two additional patients without spleno-portal abnormalities had a mild increase in transaminase levels of unclear origin. None of patients with INCPH had signs of liver failure defined as INR > 1.5 and albumin < 3.5 and none bled from varices. The only patient with large esophageal varices was submitted to endoscopic therapy as a primary prophylaxis of variceal bleeding. This procedure was effective since she had no bleeding episodes in the 3 years period after this treatment. None of patients underwent transjugular intrahepatic portosystemic shunt (TIPS). None of the patients with INCPH died from complications of portal hypertension. However, two patients with INCPH (one with CVID and one with XLA) died as a consequence of sepsis.

### 3.3. Immunological Abnormalities in PAD Patients with Isolated Splenomegaly and in Patients with INCPH and/or Portal Vein Enlargement

**Figure 4:**
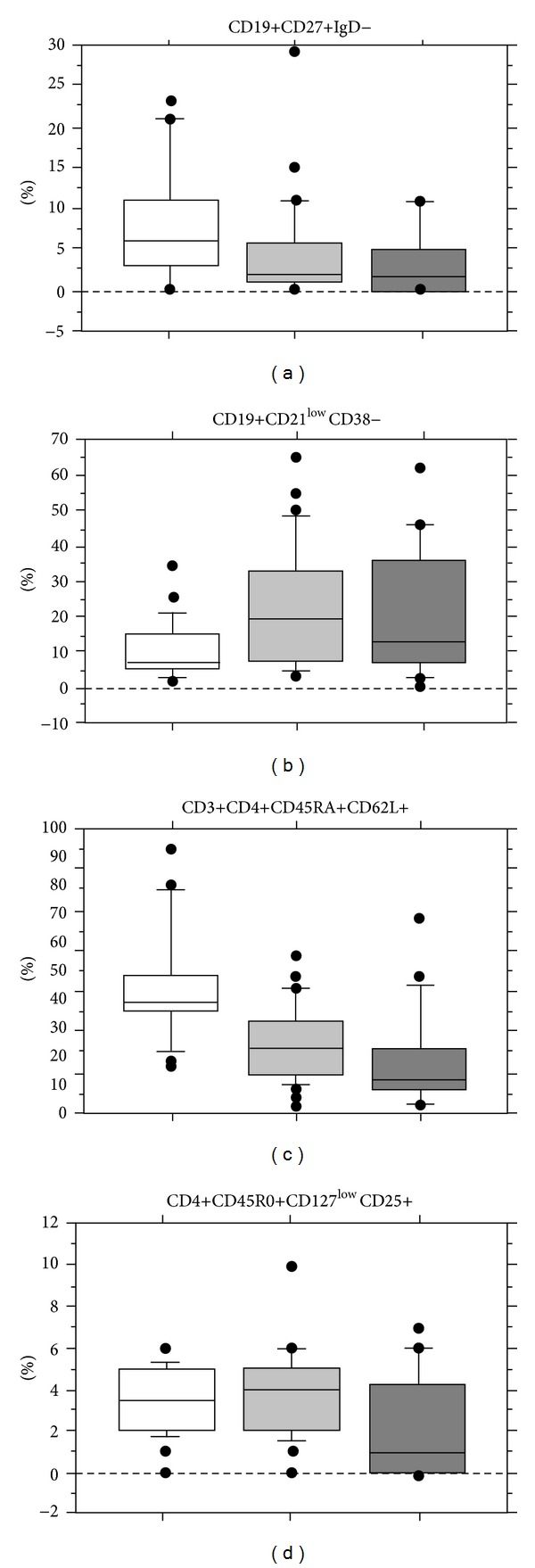
Peripheral blood lymphocyte immunophenotype in PAD patients without spleno-portal axis abnormalities (white square), with isolated splenomegaly (gray square) and abnormalities of spleno-portal axis (black square). CD19+27+IgD− (switched memory B cells), CD19+CD21 low B cells, CD3+CD4+CD45RA+ (naïve CD4+ T cells), and CD4+CD25+CD127− (regulatory T cells) are shown. Results are expressed as percentages ± SD.

We demonstrated that in comparison to PAD patients without splenomegaly PAD patients with splenomegaly have a decrease in the frequency of switched memory B cells (4.1 ± 5.4% versus 7.9 ± 6.9%; *P* = 0.01) and an increased frequency of CD21 low B cells (20.1 ± 18 versus 11.4 ± 10; *P* = 0.02) confirming our previous data [20]. Abnormalities of T cell subsets frequencies were also observed in CD3+CD4+ cells (35.17 ± 11.8% versus 42.7 ± 11.15%; *P* = 0.01), in naïve CD4+ cells (22.4 ± 15.2% versus 44.18 ± 19.1%; *P* < 0.0001), and in CD4+ memory T cells (81.2 ± 13 versus 67.45 ± 15.5; *P* = 0.0003). More profound defects in B and T cell subsets were observed in patients with INCPH and/or portal vein enlargement. In fact, immunological abnormalities were more evident when we analysed PAD patients according to their clinical phenotype: patients with spleno-portal axis abnormalities, patients with isolated splenomegaly, and patients without spleno-portal abnormalities (Figure 4): lower frequencies of switched memory B cells (3.1 ± 4.2%, versus 4.6 ± 6.2 versus 7.6 ± 6.6  *P* = 0.04) and CD4+CD45RA+CD62L+ naïve T cells (14.8 + 12.3% versus 24.6 ± 13.4 versus 44.3 ± 19.5  *P* < 0.0001) and increased frequencies of CD4+ memory T cells (89.3 ± 10% versus 78.5 ± 15.7% versus 67 ± 15.7; *P* = 0.00003). Severe abnormalities were also found in the frequencies of CD21 low cells that were increased (23.6 ± 13.4% versus 20.1 ± 18% versus 11.4 ± 10; *P* = 0.02) and in the frequencies of CD4+ regulatory T cells that were decreased (1.6 ± 2.3% versus 3.6 ± 2% versus 3.5 ± 1.6%; *P* = 0.02). The frequencies of other T and B cell subsets were comparable within groups.

Seventeen patients had less than 15% of naive CD4+ T cells, a condition recently named late onset combined immune deficiency (LOCID) [22] described in a subgroup of CVID patients at high risk of opportunistic infections. Seven out of 17 LOCID patients had portal enlargement and 2 of these had INCPH. Details of peripheral blood lymphocyte immunophenotyping with mean ± SD, median, range, percentiles, and *P* values showing comparison between the 3 groups (without splenic-axis abnormalities, with isolated splenomegaly and with spleno-portal abnormalities) were shown in Supplementary Table 2.

## 4. Discussion

This study shows that 25.6% patients with PAD had signs of portal vein enlargement but that only 1/6 of these had portal hypertension/INCPH. The subgroup of patients with INCPH or spleno-portal abnormalities without signs of portal hypertension had severe clinical and immunological phenotypes with a severe defect of B cell terminal differentiation, T cell activation, and a deficiency in regulatory T cells.

The hallmark of primary antibody deficiencies is a defect in the production of antibodies indispensable for the response against a wide variety of pathogens resulting in recurrent and severe infections. Histological abnormalities have been found in primary and secondary lymphoid organs, including spleen [19], the largest secondary lymphoid organ, and in mucosa-associated lymphoid tissues [23]. Previous studies have demonstrated that the spleen is crucial in regulating immune homoeostasis, through its ability to link innate and adaptive immunity and to protect against infections [24]. Due to its unique structure and vascularisation, spleen is also essential in the removal of various blood-borne pathogens. An increased spleen size is a common finding in PAD patients but neither its causes nor its consequences are well understood. Histological descriptions of spleen abnormalities after splenectomy showed granulomatous lesions, congestive red pulp, follicular hyperplasia, and atrophic germinal centres/white pulp [19]. In PAD, a reduction of a memory B cell subset developing in the spleen, called IgM memory cells [25], is associated with the absence of secretory IgA on intestinal epithelial cells [26]. A defect in the intestinal immunity alters the mucosal permeability associated with an increased risk of bacterial infections as it happens also in other liver diseases [27]. Both reduction of memory B cells and very low IgA levels are risk factors for various PAD-associated conditions, including respiratory and gastrointestinal infections as well as splenomegaly [20, 28, 29]. A high prevalence up to 50% of gastrointestinal symptoms, mainly chronic diarrhoea due to intestinal infections caused by* Giardia lamblia*,* Campylobacter jejuni*, or* Salmonella *spp.and malabsorption, has been described [30]. Here, we addressed the issue of the possible consequences of splenomegaly on the spleno-portal axis. We confirmed a high frequency of splenomegaly in more severe forms of PAD, CVID, and XLA [20]. About 40% of patients with splenomegaly had a portal vein enlargement or INCPH. Portal vein enlargement and INCPH developed also in patients with XLA. This is the first demonstration that portal abnormalities might occur also in XLA, further strengthening our hypothesis on INCPH pathogenesis. Splenomegaly might contribute to portal hypertension by increasing the portal flow (portal superflux). In addition, the increased frequency of intestinal infections may increase the hepatic resistance by directly affecting the portal vein branches [31]. Differently from other studies that included only PAD patients with abnormal liver function tests, we included in the study PAD patients without known causes of liver diseases. When PAD patients were selected because of functional liver abnormalities high frequencies of portal hypertension (50%) and anicteric cholestasis (65%) were observed [8]. The histological finding consisted of NRH [32] associated with lymphocytic CD8+ cytotoxic T cells intrasinusoidal infiltrate. Sinusoid lining disruption and portal vein endothelitis may imply a possible role of the intrasinusoidal infiltrating lymphocytes in the pathogenesis of the lesions. In our study at variance with previous reports [7–10] spleno-portal axis abnormalities were detected also in few PAD patients without altered liver function tests. Endoscopic band ligation to prevent the variceal bleeding was effective in the patient with large esophageal varices. This procedure should then be considered in PAD patients in agreement with the guidelines for prevention of variceal bleeding [33]. Its success further supported the Italian guidelines that recommend regularly performing an upper gastrointestinal endoscopy every two years in PAD patients (http://www.aieop.org/).

Spleno-portal axis abnormalities might be the consequences of multiple inflammatory states including autoimmunity and infections. The immune disturbance is characterized by severe B cell defects, increased numbers of exhausted CD21low B cells [34, 35], and T cell activation point to an inflammatory or infectious state. The reduced frequency of regulatory T cells might also contribute to autoimmunity and inflammation [36]. Furthermore, portal abnormalities were more frequent in the CVID group recently called LOCID, at higher risk of opportunistic infections, including gastrointestinal infections [22]. In PAD patients, infections, splenomegaly, increased blood venous flow, and inflammation, all, potentially contribute to liver damage. Chronic infections and immunological abnormalities that are the hallmark of PAD are also considered the main hypothesized pathophysiological mechanisms of INCPH [12, 37]. In PAD patients, despite the low prevalence, INCPH should be actively searched when signs of with portal hypertension are present. On the other hand, a diagnosis of PAD should be considered in patients with INCPH.

## Supplementary Material

Supplementary table 1: no need of description. the title (Detailed description of characteristics of 112 CVID and 5 XLA patients.) is autoesplicative.
Supplementary Table 2: “The analysis of immunological abnormalities in PAD patients with isolated splenomegaly and in patients with INCPH and/or portal vein enlargemen showed that in comparison to PAD patients without splenomegaly, PAD patients with splenomegaly have a decrease in the frequency of switched memory B cells and an increased frequency of CD21 low B cells. Abnormalities of T cell subsets frequencies were also observed in CD3+CD4+ cells, in naïve CD4+ cells and in CD4+ memory T cells. More profound defects in B and T cell subsets were observed in patients with INCPH and/or portal vein enlargement.”
Supplementary Figure 1: Longitudinal assessment of portal vein (A) and spleen diameter (B) by abdominal ultrasounds in 30 patients (x- axis) with spleno-axis abnormalities.
Portal and spleen diameter were measured at the diagnosis time (light gray bars), after 5 years (dark gray bars) and after 10 years (black bars). The majority of patients had already portal vein enlargement at the diagnosis time. Spleen diameter increased in the observation period.

## Figures and Tables

**Table 1:** Demographic, clinical, and laboratory characteristics of 117 PAD patients.

Female (%)	55.6	ALT (mg/dL)	24 ± 12*
Age (years, mean ± SD)	49 ± 15	AST (mg/dL)	23 ± 18*
Follow-up (years, mean ±SD)	18 ± 14	AP (mg/dL)	98 ± 63*
XLA (%)	4.2	Bilirubin (mg/dL)	0.5 ± 0.4*
CVID (%)	95.8	*γ*GT (mg/dL)	28 ± 26*
Splenomegaly (%)	60.7	INR	1 ± 0.08*
Portal vein enlargement (%)	25.6	RBC (cell/mm^3^)	4700000 ± 9430*
Esophageal varices (%)	3.4	Hb (g/dL)	13.4 ± 6*
Portal vein collaterals, other than varices (%)	0.8	WBC (cell/mm^3^)	6300 ± 2400*
Ascites (number of patients)	0	PLT (cell/mm^3^)	214000 ± 93000*
		IgG at diagnosis (mg/dL)	267 ± 175*
		IgA at diagnosis (mg/dL)	27 ± 36*
		IgM at diagnosis (mg/dL)	42 ± 79*

*All laboratory parameters are expressed as mean ± SD.

**Table 2:** Clinical data of 117 PAD patients: without spleno-portal axis abnormalities and with isolated splenomegaly and spleno-portal axis abnormalities.

	PAD with normal spleen and portal diameters (*n* = 46)	Isolated splenomegaly (*n* = 41)	PAD with spleno-portal axis abnormalities (*n* = 30)
Time of PAD disease (years)*	14.3 ± 13.8	17 ± 16.5	19.7 ± 13
Bronchiectasis	15 (33%)	13 (32%)	21 (70%)
Gastroenteritis	15 (33%)	13 (32%)	28 (93%)
Lymphoid nodular hyperplasia	4 (9%)	2 (5%)	15 (50%)
Pneumonia	28 (61%)	10 (24%)	19 (63%)
Autoimmunity	13 (28%)	6 (15%)	11 (37%)
Portal vein diameter (mm)*	10.3 ± 1.7	11 ± 1.5	14 ± 3
Spleen diameter (cm)*	10 ± 1	13 ± 2.7	14.1 ± 6.3

*Parameters are expressed as mean ± SD.
