# Supplementary material for: Idiopathic Non Cirrhotic Portal Hypertension and Spleno-Portal Axis Abnormalities in Patients with Severe Primary Antibody Deficiencies
Source: J Immunol Res. 2014 Mar 31;2014:672458. doi: 10.1155/2014/672458 (PMC3988706; doi:10.1155/2014/672458)
Supplement: Supplementary file 1 — Supplementary table 1: no need of description. the title (Detailed description of characteristics of 112 CVID and 5 XLA patients.) is autoesplicative. Supplementary Table 2: “The analysis of immunological abnormalities in PAD patients with isolated splenomegaly and in patients with INCPH and/or portal vein enlargemen showed that in comparison to PAD patients without splenomegaly, PAD patients with splenomegaly have a decrease in the frequency of switched memory B cells and an increased frequency of CD21 low B cells. Abnormalities of T cell subsets frequencies were also observed in CD3+CD4+ cells, in naïve CD4+ cells and in CD4+ memory T cells. More profound defects in B and T cell subsets were observed in patients with INCPH and/or portal vein enlargement.” Supplementary Figure 1: Longitudinal assessment of portal vein (A) and spleen diameter (B) by abdominal ultrasounds in 30 patients (x- axis) with spleno-axis abnormalities. Portal and spleen diameter were measured at the diagnosis time (light gray bars), after 5 years (dark gray bars) and after 10 years (black bars). The majority of patients had already portal vein enlargement at the diagnosis time. Spleen diameter increased in the observation period. [file 672458.f1.docx]

**Supplementary Table 1. – Detailed description of characteristics of 112 CVID and 5 XLA patients.**

CVID, common variable immunodeficiency; XLA, X linked Agammaglobulinemia; PV, portal vein; SM, splenomegaly; HM, hepatomegaly; PH, portal hypertension; EV, esophageal varices; PS, porto-systemic; A, ascites; ALT,  glutamate-oxaloacetate transaminase; AST, glutamate-pyruvate-transaminase; AP, alkaline phosphatase; GGT, gamma glutamyl transferase; ALB, albumin; PLT, platelets. *: splenectomy. 0: absence of specified sign, 1: presence of specified sign.

|  | **Patient** | **Gender** | **Age (years)** | **Disease time** | **PV enlargement** | **SM** | **HM** | **PH** | **EV** | **PS collateral** | **Ascites** | **PV(mm)** | **Spleen (cm)** | **ALT x N** | **AST X N** | **AP X N** | **GGT X N** | **INR** | **ALB (g/l)** | **Bilirubina** | **PLT(/mm3)** |
| --- | --- | --- | --- | --- | --- | --- | --- | --- | --- | --- | --- | --- | --- | --- | --- | --- | --- | --- | --- | --- | --- |
|  |  |  |  |  |  |  |  |  |  |  |  |  |  |  |  |  |  |  |  |  |  |
| **CVID** | 1 | F | 39 | 3 | 0 | 0 | 0 | 0 | 0 | 0 | 0 | 8 | 11 | X1 | X1 | X1 | X1 | 1 | 4 | 0.42 | 234000 |
|  | 2 | F | 54 | 15 | 1 | 1 | 1 | 1 | 1 | 0 | 0 | 20 | 27 | X1 | X1 | X3 | X2 | 1 | 4 | 0.35 | 65000 |
|  | 3 | F | 40 | 20 | 1 | 0 | 1 | 0 | 0 | 0 | 0 | 14 | * | X1 | X1 | X1 | X1 | 1 | 6.1 | 0.53 | 358000 |
|  | 4 | F | 52 | 2 | 0 | 1 | 0 | 0 | 0 | 0 | 0 | 10 | 12 | X1 | X1 | X1 | X1 | 1.02 | 4.2 | 0.34 | 198000 |
|  | 5 | M | 48 | 8 | 0 | 1 | 0 | 0 | 0 | 0 | 0 | 12 | 13 | X1 | X1 | X1 | X1 | 1 | 5 | 0.63 | 346000 |
|  | 6 | F | 61 | 43 | 1 | 1 | 1 | 0 | 0 | 0 | 0 | 14 | 19 | X1 | X1 | X1 | X1 | 1 | 4.8 | 0.36 | 146000 |
|  | 7 | F | 65 | 2 | 0 | 1 | 1 | 0 | 0 | 0 | 0 | 10 | 15.5 | X1 | X1 | X2 | X2 | 0.96 | 3.5 | 0.36 | 75000 |
|  | 8 | M | 42 | 16 | 0 | 1 | 0 | 0 | 0 | 0 | 0 | 11 | 13 | X1 | X1 | X1 | X1 | 1 | 4.7 | 0.44 | 201000 |
|  | 9 | F | 46 | 9 | 0 | 0 | 1 | 0 | 0 | 0 | 0 | 10 | 10 | X1 | X1 | X1 | X1 | 1 | 3.9 | 0.74 | 353000 |
|  | 10 | M | 34 | 2 | 0 | 1 | 1 | 0 | 0 | 0 | 0 | 12 | 19 | X1 | X1 | X1 | X1 | 1.04 | 4.8 | 0.6 | 116000 |
|  | 11 | F | 46 | 4 | 0 | 1 | 0 | 0 | 0 | 0 | 0 | 12 | 17 | X1 | X1 | X1 | X1 | 1 | 5 | 0.94 | 120000 |
|  | 12 | F | 68 | 20 | 0 | 1 | 0 | 0 | 0 | 0 | 0 | 11 | 13.5 | X1 | X1 | X1 | X1 | 1 | 4.2 | 0.38 | 172000 |
|  | 13 | M | 47 | 3 | 0 | 1 | 1 | 0 | 0 | 0 | 0 | 11 | 12 | X1 | X1 | X1 | X1 | 1.04 | 3.7 | 0.3 | 206000 |
|  | 14 | M | 23 | 3 | 0 | 0 | 0 | 0 | 0 | 0 | 0 | 10 | 11 | X1 | X1 | X1 | X1 | 1 | 3.6 | 0.76 | 252000 |
|  | 15 | F | 81 | 18 | 0 | 0 | 0 | 0 | 0 | 0 | 0 | 11 | 9 | X1 | X1 | X1 | X1 | 1 | 3.1 | 0.19 | 630000 |
|  | 16 | F | 64 | 7 | 1 | 1 | 1 | 0 | 0 | 0 | 0 | 17 | 22.5 | X1 | X1 | X1.5 | X1 | 1.06 | 3.3 | 0.51 | 100000 |
|  | 17 | M | 65 | 12 | 0 | 1 | 1 | 0 | 0 | 0 | 0 | 10 | 14 | X1 | X1 | X1 | X1 | 1.07 | 4.3 | 0.21 | 156000 |
|  | 18 | M | 60 | 54 | 0 | 0 | 1 | 0 | 0 | 0 | 0 | 12 | 11.5 | X1 | X1 | X1 | X1 | 0.97 | 4.4 | 1 | 151000 |
|  | 19 | M | 23 | 25 | 1 | 1 | 1 | 0 | 0 | 0 | 0 | 14 | 16 | X1 | X1 | X1 | X1 | 1 | 5.2 | 0.66 | 180000 |
|  | 20 | F | 56 | 29 | 0 | 1 | 0 | 0 | 0 | 0 | 0 | 9 | 14 | X1 | X1 | X1 | X1 | 1 | 4.5 | 0.52 | 223000 |
|  | 21 | F | 65 | 11 | 0 | 0 | 0 | 0 | 0 | 0 | 0 | 10 | 9 | X1 | X1 | X1 | X1 | 1 | 3.6 | 0.22 | 201000 |
|  | 22 | F | 44 | 15 | 0 | 0 | 1 | 0 | 0 | 0 | 0 | 10 | 11 | X1 | X1 | X1 | X1 | 1 | 4.8 | 0.45 | 233000 |
|  | 23 | F | 72 | 30 | 1 | 0 | 1 | 0 | 0 | 0 | 0 | 14 | 8 | X1 | X1 | X1 | X1 | 1 | 4.4 | 0.25 | 274000 |
|  | 24 | F | 25 | 3 | 0 | 0 | 0 | 0 | 0 | 0 | 0 | 11 | * | X1 | X1 | X1 | X1 | 1 | 4.7 | 0.34 | 530000 |
|  | 25 | F | 53 | 46 | 0 | 1 | 1 | 0 | 0 | 0 | 0 | 12 | 18 | X1 | X1 | X1 | X1 | 1.08 | 4.6 | 0.5 | 155000 |
|  | 26 | M | 37 | 0 | 0 | 0 |  | 0 | 0 | 0 | 0 | 10 | 10 | X1 | X1 | X1 | X1 | 0.93 | 4.4 | 0.36 | 176000 |
|  | 27 | M | 50 | 4 | 0 | 1 | 0 | 0 | 0 | 0 | 0 | 12 | 13 | X1 | X1 | X1 | X1 | 1 | 4.8 | 0.72 | 138000 |
|  | 28 | M | 40 |  | 0 | 1 | 0 | 0 | 0 | 0 | 0 | 12 | 14.6 | X1 | X1 | X1 | X1 | 1 | 4.7 | 0.6 | 134000 |
|  | 29 | M | 41 | 11 | 0 | 1 | 0 | 0 | 0 | 0 | 0 | 10 | 13 | X1 | X1 | X1 | X1 | 1 | 4.2 | 0.64 | 153000 |
|  | 30 | M | 46 | 7 | 0 | 1 | 0 | 0 | 0 | 0 | 0 | 9 | 12 | X1 | X1 | X1 | X1 | 1 | 3.9 | 1.96 | 73000 |
|  | 31 | F | 69 | 62 | 0 | 1 | 1 | 0 | 0 | 0 | 0 | 12 | 14 | X1 | X1 | X1 | X1 | 0.96 | 44 | 0.2 | 181000 |
|  | 32 | M | 64 | 29 | 1 | 1 | 1 | 0 | 0 | 0 | 0 | 15 | 17 | X1 | X1 | X1 | X1 | 1 | 5 | 0.53 | 114000 |
|  | 33 | F | 73 | 4 | 0 | 0 | 0 | 0 | 0 | 0 | 0 | 8 | 11 | X1 | X1 | X1 | X1 | 1.11 | 4.8 | 0.39 | 212000 |
|  | 34 | F | 50 | 4 | 0 | 0 | 1 | 0 | 0 | 0 | 0 | 11 | 10 | X1 | X1 | X1 | X1 | 1 | 4 | 0.36 | 268000 |
|  | 35 | M | 32 | 20 | 1 | 1 | 0 | 0 | 0 | 0 | 0 | 14 | 13.3 | X1 | X1 | X1 | X1 | 1.05 | 4.8 | 1.06 | 136000 |
|  | 36 | F | 72 | 15 | 0 | 0 | 1 | 0 | 0 | 0 | 0 | 9 | 9 | X1 | X1 | X1 | X1 | 1.1 | 5.6 | 0.6 | 221000 |
|  | 37 | F | 65 | 11 | 0 | 0 | 0 | 0 | 0 | 0 | 0 | 10 | 10 | X1 | X1 | X1 | X1 | 1 | 2.4 | 0.4 | 257000 |
|  | 38 | F | 37 | 3 | 0 | 1 | 0 | 0 | 0 | 0 | 0 | 10 | 12 | X1 | X1 | X1 | X1 | 1.02 | 4.9 | 0.19 | 195000 |
|  | 39 | F | 35 | 1 | 0 | 1 | 0 | 0 | 0 | 0 | 0 | 9 | 12 | X1 | X1 | X1 | X1 | 1 | 4 | 0.35 | 179000 |
|  | 40 | F | 52 | 4 | 0 | 0 | 1 | 0 | 0 | 0 | 0 | 10 | 9 | X1 | X1 | X1 | X1 | 0.9 | 5 | 0.53 | 279000 |
|  | 41 | M | 55 | 55 | 1 | 1 | 1 | 0 | 0 | 0 | 0 | 18 | 25 | X1 | X1 | X1 | X1 | 1 | 4.3 | 0.7 | 111000 |
|  | 42 | M | 39 | 20 | 0 | 1 | 1 | 0 | 0 | 0 | 0 | 11 | 16 | X1 | X1 | X1 | X1 | 0.9 | 4.6 | 0.6 | 149000 |
|  | 43 | F | 60 | 25 | 1 | 1 | 1 | 1 | 0 | 1 | 0 | 17 | 18.5 | X1 | X1 | X2 | X1.5 | 1.04 | 4.4 | 0.69 | 86000 |
|  | 44 | M | 60 | 18 | 1 | 1 | 1 | 0 | 0 | 0 | 0 | 14 | 13 | X1 | X1 | X1 | X1 | 0.9 | 4 | 0.38 | 273000 |
|  | 44 | F | 40 |  | 0 | 0 | 0 | 0 | 0 | 0 | 0 | 10 | 9 | X1 | X1 | X1 | X1 | 1 | 4.2 | 0.5 | 293000 |
|  | 46 | F | 37 | 32 | 0 | 0 | 1 | 0 | 0 | 0 | 0 | 11 | 9.5 | X1 | X1 | X1 | X1 | 1 | 4.3 | 0.63 | 254000 |
|  | 47 | M | 68 | 9 | 0 | 0 | 0 | 0 | 0 | 0 | 0 | 12 | 11 | X1 | X1 | X1 | X1 | 1 | 4.2 | 0.44 | 157000 |
|  | 48 | F | 60 | 3 | 0 | 0 | 0 | 0 | 0 | 0 | 0 | 9 | * | X1 | X1 | X1 | X1 | 1.05 | 4.44 | 0.43 | 201000 |
|  | 49 | F | 64 | 35 | 1 | 1 | 0 | 0 | 0 | 0 | 0 | 15 | 14 | X1 | X1 | X1 | X1 | 1 | 3.42 | 0.46 | 155000 |
|  | 50 | M | 38 |  | 0 | 1 | 0 | 0 | 0 | 0 | 0 | 9 | 12 | X1 | X1 | X1 | X1 | 1 | 4.6 | 0.74 | 206000 |
|  | 51 | F | 37 | 2 | 0 | 1 | 0 | 0 | 0 | 0 | 0 | 12 | 12.5 | X1 | X1 | X1 | X1 | 1 | 4.7 | 1.08 | 144000 |
|  | 52 | M | 44 | 16 | 0 | 1 | 1 | 0 | 0 | 0 | 0 | 10 | 16 | X1 | X1 | X1 | X1 | 0.98 | 4.5 | 0.3 | 158000 |
|  | 53 | M | 50 | 11 | 1 | 1 | 1 | 0 | 0 | 0 | 0 | 14 | 15 | X1 | X1 | X1 | X1 | 1.03 | 4.7 | 1.53 | 212000 |
|  | 54 | F | 54 | 23 | 0 | 0 | 0 | 0 | 0 | 0 | 0 | 9 | 9 | X1 | X1 | X1 | X1 | 1 | 4.2 | 0.36 | 249000 |
|  | 55 | M | 45 | 23 | 1 | 1 | 1 | 1 | 1 | 0 | 0 | 22.9 | 29.4 | X1 | X1 | X1,5 | X1 | 1.08 | 4.4 | 0.32 | 48000 |
|  | 56 | M | 40 |  | 0 | 1 | 0 | 0 | 0 | 0 | 0 | 12 | 16 | X1 | X1 | X1 | X1 | 1 | 4.6 | 1 | 171000 |
|  | 57 | F | 39 | 2 | 1 | 1 | 0 | 0 | 0 | 0 | 0 | 14 | 12.5 | X1 | X1 | X1 | X1 | 1.06 | 4.6 | 0.36 | 205000 |
|  | 58 | F | 65 | 4 | 1 | 0 | 0 | 0 | 0 | 0 | 0 | 14 | 9 | X1 | X1 | X1 | X1 | 1 | 4.1 | 0.47 | 225000 |
|  | 59 | M | 28 | 8 | 1 | 1 | 1 | 0 | 0 | 0 | 0 | 14 | 16 | X1 | X1 | X1 | X1 | 1.2 | 4.2 | 0.57 | 81000 |
|  | 60 | F | 53 | 53 | 0 | 0 | 0 | 0 | 0 | 0 | 0 | 8 | 7.4 | X1 | X1 | X1 | X1 | 1 | 3.6 | 0.54 | 510000 |
|  | 61 | F | 55 | 6 | 0 | 0 | 0 | 0 | 0 | 0 | 0 | 9 | 8 | X1 | X1 | X1 | X1 | 1 | 3.8 | 0.36 | 234000 |
|  | 62 | F | 32 | 2 | 0 | 0 | 0 | 0 | 0 | 0 | 0 | 11 | 10 | X1 | X1 | X1 | X1 | 1 | 4 | 0.2 | 172000 |
|  | 63 | F | 39 | 4 | 0 | 1 | 0 | 0 | 0 | 0 | 0 | 8 | 12 | X1 | X1 | X1 | X1 | 1 | 4.3 | 0.22 | 253000 |
|  | 64 | M | 48 | 13 | 0 | 1 | 0 | 0 | 0 | 0 | 0 | 11 | 16 | X1 | X1 | X2.5 | X1 | 1 | 5 | 0.37 | 159000 |
|  | 65 | F | 42 | 4 | 0 | 1 | 0 | 0 | 0 | 0 | 0 | 11 | 13 | X1 | X1 | X1 | X1 | 1 | 4,5 | 0,31 | 194000 |
|  | 66 | M | 29 | 15 | 1 | 1 | 0 | 0 | 0 | 0 | 0 | 14 | 13.5 | X1 | X1 | X1 | X1 | 1 | 4 | 0.55 | 377000 |
|  | 67 | F | 44 | 2 | 0 | 0 | 0 | 0 | 0 | 0 | 0 | 9 | 9 | X1 | X1 | X1 | X1 | 0.93 | 4.3 | 0.32 | 283000 |
|  | 68 | F | 53 | 42 | 0 | 1 | 1 | 0 | 0 | 0 | 0 | 11 | 12 | X1 | X1 | X1 | X1 | 1 | 4 | 0.82 | 218000 |
|  | 69 | F | 53 | 36 | 0 | 0 | 0 | 0 | 0 | 0 | 0 | 10 | 11 | X1 | X1 | X1 | X1 | 1 | 4.3 | 0.48 | 145000 |
|  | 70 | F | 56 | 5 | 0 | 0 | 0 | 0 | 0 | 0 | 0 | 9 | 10 | X1 | X1 | X1 | X1 | 0.97 | 4.4 | 0.25 | 155000 |
|  | 71 | M | 25 | 7 | 1 | 1 | 1 | 1 | 1 | 0 | 0 | 14.2 | 20 | X3 | X4.5 | X1 | X1 | 1.08 | 4.6 | 0.6 | 170000 |
|  | 72 | M | 43 | 21 | 1 | 1 | 0 | 0 | 0 | 0 | 0 | 14 | 12.5 | X1 | X1 | X1 | X1 | 1 | 4.3 | 0.33 | 238000 |
|  | 73 | F | 62 | 54 | 0 | 0 | 1 | 0 | 0 | 0 | 0 | 10 | * | X1 | X1 | X1.5 | X1 | 1 | 2 | 0.45 | 253000 |
|  | 74 | M | 45 | 30 | 1 | 1 | 0 | 0 | 0 | 0 | 0 | 14 | 14 | X1 | X1 | X1 | X1 | 0.9 | 3.4 | 0.33 | 197000 |
|  | 75 | F | 52 | 2 | 1 | 0 | 0 | 0 | 0 | 0 | 0 | 14 | 10 | X1 | X1 | X1 | X1 | 0.88 | 4.7 | 0.32 | 145000 |
|  | 76 | M | 21 | 11 | 0 | 1 | 0 | 0 | 0 | 0 | 0 | 9 | 13 | X1 | X1 | X1 | X1 | 1.08 | 4.8 | 0.98 | 291000 |
|  | 77 | F | 57 | 44 | 0 | 1 | 0 | 0 | 0 | 0 | 0 | 11 | 13 | X1 | X1 | X1 | X1 | 0.97 | 4 | 0.3 | 140000 |
|  | 78 | F | 28 | 11 | 0 | 0 | 0 | 0 | 0 | 0 | 0 | 11 | 11 | X1 | X1 | X1 | X1 | 1.09 | 4.5 | 0.38 | 234000 |
|  | 79 | F | 61 | 11 | 0 | 0 | 0 | 0 | 0 | 0 | 0 | 11 | 10 | X1 | X1 | X1 | X1 | 0.98 | 4.1 | 0.31 | 164000 |
|  | 80 | F | 49 | 44 | 0 | 0 | 0 | 0 | 0 | 0 | 0 | 11 | 11 | X1 | X1 | X1 | X1 | 1,02 | 4.7 | 0.32 | 259000 |
|  | 81 | F | 50 | 4 | 0 | 0 | 0 | 0 | 0 | 0 | 0 | 10 | 11 | X1 | X1 | X1 | X1 | 1 | 4.4 | 0.12 | 221000 |
|  | 82 | F | 57 | 1 | 0 | 0 | 1 | 0 | 0 | 0 | 0 | 7 | 10 | X1 | X1 | X1 | X1 | 1 | 4.7 | 0.24 | 222000 |
|  | 83 | F | 32 | 16 | 0 | 1 | 1 | 0 | 0 | 0 | 0 | 11 | 13 | X1 | X1 | X1 | X1 | 1 | 4.4 | 0.25 | 146000 |
|  | 84 | M | 21 | 2 | 1 | 1 | 1 | 0 | 0 | 0 | 0 | 14 | 17 | X1 | X1 | X1 | X1 | 1.1 | 5 | 0.8 | 96000 |
|  | 85 | F | 38 | 23 | 0 | 1 | 0 | 0 | 0 | 0 | 0 | 11 | 14 | X1 | X1 | X1 | X1 | 0.98 | 4.3 | 0.38 | 290000 |
|  | 86 | F | 23 | 10 | 0 | 1 | 1 | 0 | 0 | 0 | 0 | 10 | 14 | X1 | X1 | X1 | X1 | 1.08 | 4.4 | 0.97 | 167000 |
|  | 87 | F | 59 | 26 | 1 | 1 | 0 | 0 | 0 | 0 | 0 | 14 | 13 | X1 | X1 | X1 | X1 | 1.11 | 4.5 | 0.42 | 178000 |
|  | 88 | M | 32 | 21 | 0 | 0 | 0 | 0 | 0 | 0 | 0 | 12 | 11 | X1 | X1 | X1 | X1 | 1 | 4.7 | 0.62 | 199000 |
|  | 89 | M | 28 | 25 | 0 | 0 | 0 | 0 | 0 | 0 | 0 | 10 | 10 | X1 | X1 | X1 | X1 | 1 | 4.8 | 0.48 | 175000 |
|  | 90 | M | 32 | 2 | 0 | 1 | 1 | 0 | 0 | 0 | 0 | 10 | 13 | X1 | X1 | X1 | X1 | 1.04 | 4.7 | 0.44 | 215000 |
|  | 91 | M | 40 | 20 | 0 | 1 | 0 | 0 | 0 | 0 | 0 | 11 | 14 | X1 | X1 | X1 | X1 | 1.4 | 5 | 3.09 | 162000 |
|  | 92 | M | 68 | 6 | 1 | 0 | 0 | 0 | 0 | 0 | 0 | 13 | 11 | X1 | X1 | X1 | X1 | 1.04 | 4.6 | 1.15 | 209000 |
|  | 93 | M | 41 | 1 | 1 | 0 | 1 | 0 | 0 | 0 | 0 | 14 | 11 | X1 | X1 | X1 | X1 | 1.08 | 4.8 | 1.34 | 153000 |
|  | 94 | M | 59 | 6 | 0 | 1 | 1 | 0 | 0 | 0 | 0 | 12 | 13 | X1 | X1 | X1 | X1 | 0.94 | 4.5 | 0.37 | 343000 |
|  | 95 | M | 75 | 5 | 0 | 0 | 0 | 0 | 0 | 0 | 0 | 11 | 11 | X1 | X1 | X1 | X1 | 0.93 | 3 | 0.51 | 162000 |
|  | 96 | F | 38 | 3 | 0 | 1 | 0 | 0 | 0 | 0 | 0 | 10 | 13.5 | X1 | X1 | X1 | X1 | 0.99 | 5 | 0.54 | 196000 |
|  | 97 | F | 68 | 3 | 0 | 1 | 0 | 0 | 0 | 0 | 0 | 12 | 12 | X1 | X1 | X1 | X1 | 0.97 | 4 | 0.6 | 177000 |
|  | 98 | F | 48 | 4 | 0 | 0 | 0 | 0 | 0 | 0 | 0 | 8 | 10 | X1 | X1 | X1 | X1 | 0.83 | 3.9 | 0.5 | 268000 |
|  | 99 | F | 71 | 8 | 1 | 0 | 1 | 0 | 0 | 0 | 0 | 16 | * | X1 | X1 | X1 | X1 | 1.05 | 3.9 | 0.78 | 296000 |
|  | 100 | M | 73 | 7 | 0 | 0 | 0 | 0 | 0 | 0 | 0 | 12 | 11 | X1 | X1 | X1 | X1 | 1 | 4.6 | 0.52 | 172000 |
|  | 101 | F | 85 | 45 | 0 | 1 | 0 | 0 | 0 | 0 | 0 | 9 | 13 | X1 | X1 | X1.5 | X1 | 0.88 | 4 | 0.4 | 186000 |
|  | 102 | M | 65 | 6 | 0 | 0 | 0 | 0 | 0 | 0 | 0 | 11 | 11.3 | X1 | X1 | X1 | X1 | 1.07 | 4.4 | 0.8 | 238000 |
|  | 103 | F | 49 | 45 | 0 | 1 | 1 | 0 | 0 | 0 | 0 | 8 | 12 | X1 | X1 | X1 | X1 | 0.99 | 4.6 | 0.19 | 409000 |
|  | 104 | F | 36 | 33 | 0 | 0 | 0 | 0 | 0 | 0 | 0 | 10 | 9 | X1 | X1.5 | X1 | X1 | 0.96 | 3.2 | 0.24 | 462000 |
|  | 105 | F | 51 | 34 | 1 | 1 | 1 | 0 | 0 | 0 | 0 | 15 | 13 | X1 | X1 | X1 | X1 | 1 | 3.4 | 0.59 | 136000 |
|  | 106 | F | 73 | 23 | 0 | 0 | 0 | 0 | 0 | 0 | 0 | 9 | 11 | X1 | X1 | X1 | X1 | 0.99 | 4.4 | 0.3 | 201000 |
|  | 107 | M | 42 | 5 | 0 | 0 | 0 | 0 | 0 | 0 | 0 | 12 | 10 | X1 | X1 | X1 | X1 | 1 | 4.8 | 1.19 | 296000 |
|  | 108 | M | 43 | 24 | 0 | 0 | 0 | 0 | 0 | 0 | 0 | 11 | 8 | X1 | X1 | X1 | X1 | 1 | 5 | 0.42 | 187000 |
|  | 109 | M | 57 | 2 | 0 | 0 | 0 | 0 | 0 | 0 | 0 | 8 | 9 | X1 | X1 | X1 | X1 | 1 | 4.5 | 1.24 | 304000 |
|  | 110 | M | 37 | 32 | 0 | 0 | 0 | 0 | 0 | 0 | 0 | 11 | 11 | X1 | X1 | X1 | X1 | 1 | 4.5 | 0.74 | 140000 |
|  | 111 | F | 85 | 28 | 0 | 0 | 0 | 0 | 0 | 0 | 0 | 12 | 10 | X1 | X1 | X1 | X1 | 1.06 | 4.2 | 0.6 | 118000 |
| **XLA** | 112 | M | 38 | 38 | 0 | 1 | 1 | 0 | 0 | 0 | 0 | 12 | 13.5 | X1 | X1 | X1 | X1 | 1.2 | 3.9 | 0.4 | 307000 |
|  | 113 | M | 33 | 33 | 0 | 1 | 1 | 0 | 0 | 0 | 0 | 12 | 13 | X1 | X1 | X1 | X1 | 1.07 | 4.3 | 0.8 | 340000 |
|  | 114 | M | 17 | 17 | 0 | 0 | 0 | 0 | 0 | 0 | 0 | 10 | 9 | X1 | X1 | X1 | X1 | 1.13 | 4 | 0.22 | 271000 |
|  | 115 | M | 22 | 22 | 0 | 0 | 0 | 0 | 0 | 0 | 0 | 11 | 11 | X1 | X1.5 | X1 | X1 | 1 | 3.9 | 0.18 | 347000 |

|  | 116 | M | 40 | 40 | 1 | 1 | 0 | 0 | 0 | 0 | 0 | 14 | 14 | X1 | X1 | X1 | X1 | 1.12 | 4 | 1.37 | 219000 |
| --- | --- | --- | --- | --- | --- | --- | --- | --- | --- | --- | --- | --- | --- | --- | --- | --- | --- | --- | --- | --- | --- |
|  | 117 | M | 40 | 40 | 1 | 1 | 1 | 1 | 1 | 0 | 0 | 17 | 19 | X1 | X1 | X2 | X1.5 | 0.91 | 4.5 | 0.22 | 303000 |

|  |
| --- |
|  |
|  |

|  | **Patient** | **Gender** | **Age (years)** | **Disease time** | **PV enlargement** | **SM** | **HM** | **PH** | **EV** | **PS collateral** | **Ascites** | **PV(mm)** | **Spleen (cm)** | **ALT x N** | **AST X N** | **AP X N** | **GGT X N** | **INR** | **ALB (g/l)** | **Bilirubina** | **PLT(/mm3)** |
| --- | --- | --- | --- | --- | --- | --- | --- | --- | --- | --- | --- | --- | --- | --- | --- | --- | --- | --- | --- | --- | --- |
|  |  |  |  |  |  |  |  |  |  |  |  |  |  |  |  |  |  |  |  |  |  |

**Supplementary Table 2.**

**Lymphocyte subsets in 64 PAD patients with or without spleen enlargement or portal vein enlargement**

|  | **Patients without splenic-axis abnormalities** | | | | **Patients with isolated splenomegaly** | | | | **Patients with spleno-portal abnormalities** | | | | **Reference range** | **p** |
| --- | --- | --- | --- | --- | --- | --- | --- | --- | --- | --- | --- | --- | --- | --- |
|  | **(n. 21)** | | | | **(n. 26)** | | | | **(n.17)** | | | | **(controls)** |  |
|  | *mean±SD* | *percentile* | *range (mininum-maximum)* | *median* | *mean±SD* | *percentile* | *range (mininum-maximum)* | *median* | *mean±SD* | *percentile* | *range (mininum-maximum)* | *median* |  |  |
| **Lymphocytes/mm3** | **2008 ± 811** | **87** | **640-3620** | **1785** | **1738 ± 827** | **57** | **480-3644** | **1590** | **1444 ± 595** | **27** | **280-2650** | **1495** | **1000-4800** | ***NS*** |
| **CD19+ B Cells (%)** | **12.8 ± 8.1** | **70** | **1-39** | **11** | **9.5 ± 7.9** | **48** | **0,3-25** | **9** | **8.3 ± 7.9** | **43** | **0-38** | **9** | **4,9-18,4** | ***NS*** |
| **Naive B cells** (*%)* ***(CD19+CD27-IgD+)*** | **65 ± 22** | **30** | **4-95** | **65,5** | **73.2 ± 21** | **33** | **23-97** | **77,5** | **74.7 ±1 8** | **49** | **0-97** | **76,5** | **42,6-82,3** | ***NS*** |
| **IgM memory B cells** (%) ***(CD19+CD27+IgD+)*** | **23.1 ± 20** | **68** | **1-89** | **17** | **15.5 ± 16** | **55** | **1-68** | **9** | **14.5 ± 12** | **52** | **0-47** | **12** | **7,4-32,5** | ***NS*** |
| **Switched memory B cells** (%)  ***(CD19+ CD27+ IgD-)*** | **7.6 ± 6.6** | **75** | **0-23** | **6** | **4.6 ± 6.2** | **55** | **0-29** | **2** | **3,1 ± 4.2** | **30** | **0-11** | **1,5** | **6,5-29,1** | **0,04** |
| **Transitional B cells** (%) **(*CD19+CD38hiIgMhi)*** | **5.4 ± 5.0** | **58** | **0-41** | **2** | **5.3 ± 5.0** | **57** | **0-24** | **4** | **5.3 ± 4.8** | **57** | **0-30** | **4,5** | **0,6-3,4** | ***NS*** |
| **CD21low B cells** (%) ***(CD19+CD21low CD38-)*** | **11.4 ± 10** | **48** | **2-34** | **7** | **20.1 ± 18** | **68** | **3-65** | **19,5** | **23.6 ± 17** | **60** | **0-62** | **13** | **0,9-7,6** | **0,02** |
| **CD3+T cells** (%) | **73.3 ± 9.3** | **49** | **46-91** | **75** | **76.0 ± 10** | **50** | **48-94** | **75** | **76.6 ± 12** | **51** | **56-90** | **74** | **55-83** | ***NS*** |
| **CD3+ CD4+ T cells** (%) | **41.8 ± 11** | **60** | **28-66** | **40** | **34.6 ± 11.4** | **40** | **21-58** | **32** | **36.6 ± 12.8** | **49** | **17-60** | **37** | **28-57** | ***NS*** |
| **CD3+ CD8+ T cells** (%) | **31 ± 10** | **40** | **11-48** | **30,5** | **38.4 ± 14*** | **53** | **15-67** | **39** | **37.6 ±15.4** | **51** | **19-71** | **34** | **10-39** | ***NS*** |
| **Double negative T cells** (%)  ***(CD3+ CD4- CD8-α/β TCR+)*** | **1.8 ± 1.5** | **55** | **0-8** | **1,5** | **1.9 ± 1.7** | **75** | **0-8** | **2** | **1.9 ± 1.8** | **75** | **0-3** | **1** | **0.4-2.2** | **0,02** |
| **CD4+ memory T cells** (%) ***(CD3+CD4+CD45R0+)*** | **67 ± 15.7** | **25** | **26-92** | **71,5** | **78.5 ± 11.5** | **50** | **56-97** | **80** | **89.3 ± 10** | **77** | **45-99** | **91** | **29-63** | **0,0003** |
| **CD4+ naive T cells** (%) ***(CD3+ CD4+ CD45RA+ CD62L+)*** | **44.3 ± 19.5** | **80** | **17-93** | **39** | **24.6 ± 13.4** | **40** | **3-55** | **23** | **14.8 ± 12.3** | **25** | **3-69** | **12** | **41-79** | **<0,0001** |
| **Late CD8+ effector T cells** (%)  ***(CD3+ CD8+ CD27- CD28-)*** | **36.3 ± 19** | **28** | **0-61** | **38** | **41 ± 17.8** | **40** | **7-66** | **47** | **50.4 ± 17** | **55** | **22-75** | **52** | **1-41** | ***NS*** |
| **CD8+ early effector T cells** (%)  ***(CD3+ CD8+ CD27+ CD28-)*** | **18 ± 8.8** | **40** | **6-46** | **16,5** | **23.5 ± 9.5** | **61** | **11-55** | **21,5** | **19.2 ± 7.7** | **50** | **7-34** | **19** | **3-18** | ***NS*** |
| **CD4+ reg T cells** (%) **(**CD4+ CD45R0+ CD127low CD25+) | **3.5 ± 1.6** | **60** | **0-6** | **3,5** | **3.6 ± 2** | **60** | **0-10** | **4** | **1.6 ± 2.3** | **23** | **0-7** | **1** | **1.4-5.1** | **0,02** |
| **NK cells (%)**  ***(CD3- CD16+ CD56+)*** | **8.5 ± 4.6** | **60** | **3-26** | **7,5** | **8.5 ± 6.7** | **60** | **1-36** | **8** | **8.5 ± 6.3** | **60** | **2-18** | **6** | **3-10** | ***NS*** |


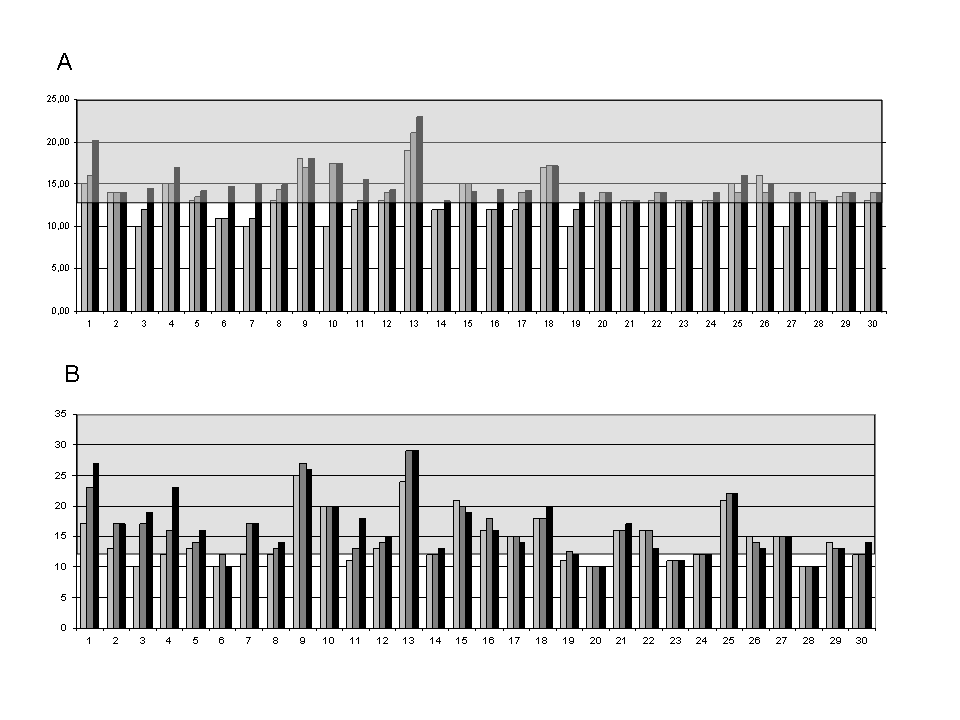


**Supplementary Fig. 1**
